# Four Letters with Answers

**Published:** 1878-01

**Authors:** 


### Our Correspondence.

CUBA, N. Y., Oct. 18, 1877.

DR. UP DE GRAFF:

Can you inform me how I may know an ovarian tumor from any other abdominal tumor? * * *

M.

Consult a competent surgeon who can give you the desired information. In the meantime you might read quite a full article upon this subject in the BISTOURY for April, 1874.

---

SOMERVILLE, Mass., Nov. 16, 1877.

DR. UP DE GRAFF:

Please send us your wide-awake BISTOURY. Its suggestions are practical. I wish its columns would warn against the use of such preparations for the hair as Ayer's Hair Vigor. The horrible ringing sounds in my head have disappeared as soon as I stopped its use, but the numbness in my limbs continue. Can you suggest a remedy to its many victims? MRS. F. H.

The above mentioned nostrum contains 0.6 per centum of sugar of lead, which, when applied to the scalp, is liable to produce the symptoms complained of. We have seen numerous cases of lead poisoning produced by the use of the various "hair tonics" advertised in the public print. We do not know of any that are safe to use or that will, in any manner, benefit the user.

---

ANDOVER, N. Y., Dec. 8, 1877.

DR. UP DE GRAFF:

Can you tell me what "Dr. Pierce's Golden Medical Discovery" is made of? I have been taking a good deal of it lately, and am growing worse all the while. My doctor says the patent medicine is the cause of the trouble. * * * *

J. D.

Any man that will swallow *any* sort of a secret nostrum, in our opinion, should be sick, and grow worse, too.

An analysis of the nostrum referred to causes a one-dollar bottle to hold 220 grains of a brownish colored, clear liquid, consisting of 15 grs. of pure honey, 1 gr. of extract of poisonous or acrid lettuce (lactuca sorosa), 2 grs. of laudanum, 100 grs. of diluted alcohol, with 105 grs. of water.

Drink away at it, and have a good time.

---

WAVERLY, Dec. 10, 1877.

DR. UP DE GRAFF:

We are having a terrible time with diphtheria. Can you send me a BISTOURY containing the most recent treatment for it?

See article in present number.

